# Protocol for engineering binding domains to recognize ligand-bound receptors by using yeast surface display

**DOI:** 10.1016/j.xpro.2024.103339

**Published:** 2024-09-24

**Authors:** Markus Dobersberger, Delia Sumesgutner, Charlotte U. Zajc, Michael W. Traxlmayr

**Affiliations:** 1 Department of Chemistry, Institute of Biochemistry, BOKU University, Vienna 1190, Austria; 2 CD Laboratory for Next Generation CAR T Cells, Vienna 1090, Austria

**Keywords:** flow cytometry, cancer, biotechnology and bioengineering

## Abstract

Yeast surface display is a versatile protein engineering technology, enabling precise control of the applied selection pressure. We present a yeast-surface-display-based protocol for the enrichment of binders specifically recognizing ligand-bound receptors. We describe steps for magnetic bead selections, random mutagenesis, and flow cytometric sorting, followed by library sequencing and detailed analysis of enriched clones. While this approach is exemplified with rcSso7d-based libraries and epidermal growth factor (EGF)-epidermal growth factor receptor (EGFR) complexes, it can also be adapted to other binder scaffolds and ligand-receptor systems.

For complete details on the use and execution of this protocol, please refer to Dobersberger et al.^1^

## Highlights

• Yeast-display-based protocol for engineering binders against activated receptors
• Steps for enrichment of binding domains via magnetic bead selection and flow sorting
• Instructions for plasmid isolation, error-prone PCR, and single-clone analysis
• Also applicable to other activated receptor conformations

## Abstract (editor highlights)

Publisher’s note: Undertaking any experimental protocol requires adherence to local institutional guidelines for laboratory safety and ethics.

## Graphical abstract

**Figure:**
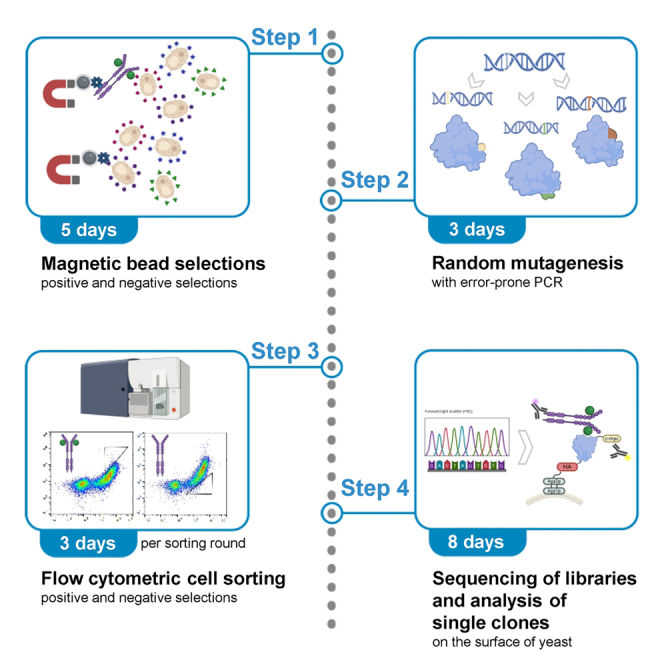

## Before you begin

Yeast surface display is a powerful protein engineering technology, enabling the expression of randomly mutated protein libraries on yeast cells and selection for desired properties, such as antigen binding. In the most widely used system, the protein of interest is expressed as an Aga2p-fusion protein on the yeast surface.^2^^,^^3^^,^^4^ Since the yeast cells also contain a plasmid encoding the mutated protein that is displayed on the surface, enriched yeast clones can be further expanded, followed by plasmid isolation and sequencing. Yeast surface display has been employed for a range of applications, including *de novo* binder engineering,^5^^,^^6^^,^^7^ affinity maturation,^6^^,^^8^ stability engineering^9^^,^^10^^,^^11^ and epitope mapping,^12^ among others.

In this protocol, we describe a yeast surface display-based approach to enrich binding domains that are specific for a ligand-receptor complex through a combination of magnetic bead selections and flow cytometric sorting. The described experimental procedure is based on standard yeast display protocols,^2^^,^^13^ which were adapted to be able to specifically target ligand-activated receptors. The key feature in our approach is a scheme of alternating positive and negative selections, i.e., selections for binding to the ligand-receptor complex and for non-binding to the ligand-free receptor state, respectively. After several rounds of selection, single clones are isolated and characterized. As previously described in Dobersberger et al.,^1^ we have used the extracellular domain of the epidermal growth factor receptor (EGFR) fused to human immunoglobulin G1 (IgG1)-Fc (termed EGFR-Fc) as soluble antigen in combination with the EGFR ligand EGF. However, this protocol can also be applied to other ligand-receptor complexes, provided that the antigen is natively folded.

Furthermore, while in this protocol the binder scaffold rcSso7d (reduced charge Sso7d^8^) is used exemplarily, this experimental approach can easily be adapted to any other yeast display library that is available, such as those based on single chain variable fragments (scFvs),^5^^,^^14^ Sso7d^15^ (from which rcSso7d was derived) or the tenth type III domain of human fibronectin (Fn3),^6^^,^^16^ among others.

### Biotinylation of the antigen

**Timing: 3 h**

**Figure 1:**
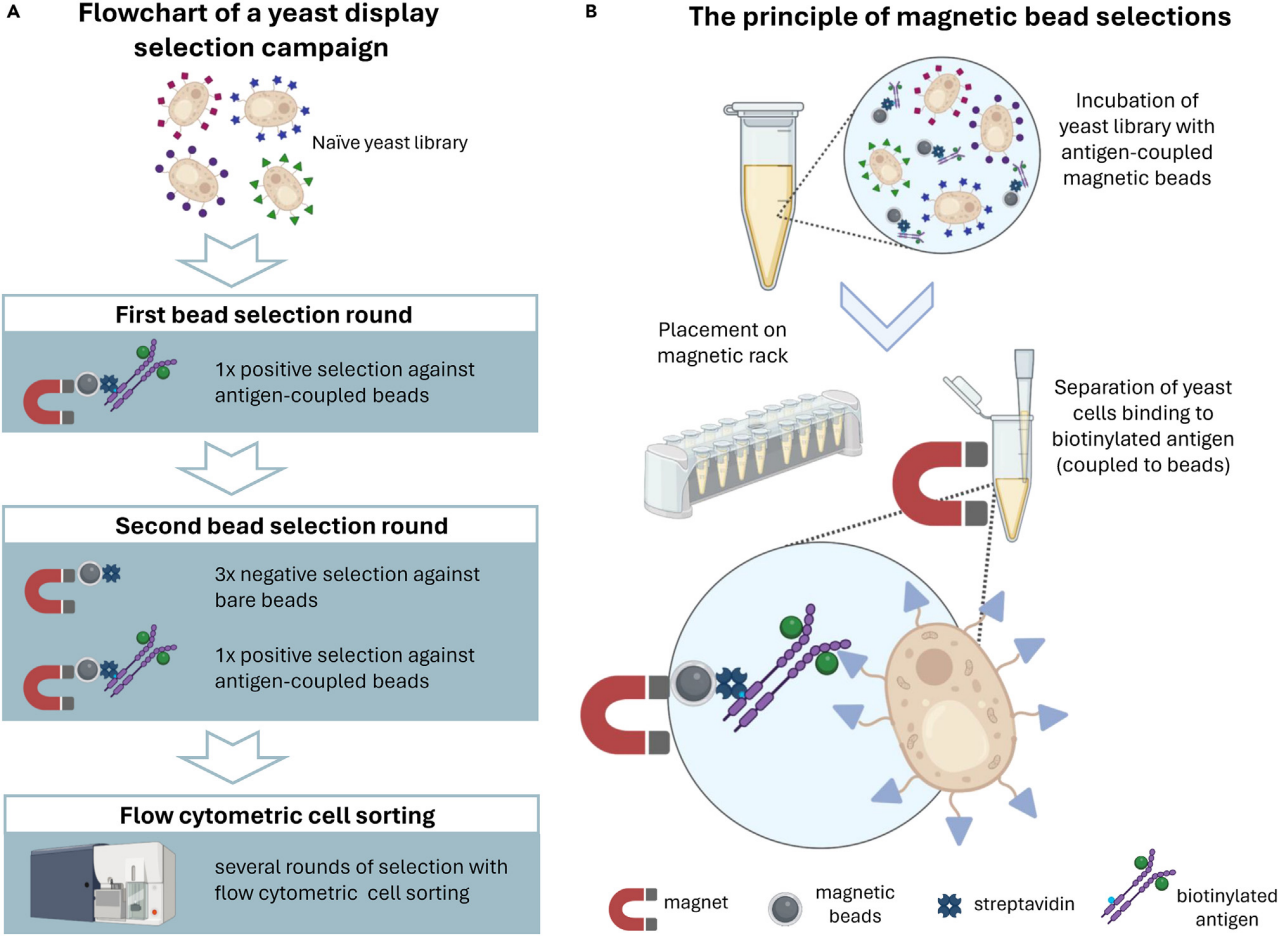
Scheme of a yeast display selection campaign and the underlying principle of magnetic bead selections (A) Workflow of yeast display selection campaigns involving magnetic bead selections and flow cytometric cell sorting. The naïve yeast library is subjected to a first positive selection with magnetic bead sorting. For this purpose, biotinylated antigen (here: ligand–receptor complex) is captured on the magnetic streptavidin beads. After an initial first positive selection round and enrichment of separated yeast cells, a second selection round is conducted. This comprises 3 rounds of negative selection against bare streptavidin-beads and 1 round of positive selection. After separation and enrichment of yeast cells, several rounds of flow cytometric cell sorting (positive and negative sorting rounds) are conducted. (B) The principle of magnetic bead sorting. After incubation of the antigen-loaded beads with the yeast library, the reaction tubes are placed on a magnetic rack. In case of a positive selection (selection against ligand–receptor complex), the yeast cells that are not binding to the beads are carefully discarded and the bead-bound cells are washed and further cultivated for enrichment. Figures were generated with Biorender.

The first part of this protocol involves selections with streptavidin-coated magnetic beads (Dynabeads Biotin Binder) and biotinylated antigen (Figure 1). For this purpose, the antigen needs to be biotinylated for immobilization on the beads. However, to avoid selection of binders against biotinylated epitopes, it is recommended to also have non-biotinylated antigen available and select against non-biotinylated antigen in later flow cytometric sorting rounds (Figure 1). Troubleshooting 1.

1. Centrifuge the thawed antigen (9000 *g*, 10 min, 4°C) to remove large aggregates and transfer the supernatant to a new tube.

***Note:*** The protein needs to be in an amine free buffer (e.g. PBS), as the NHS-activated biotin reagent reacts with primary amino groups.

***Note:*** Usually, 1 mL of antigen at a concentration of 1 μM is sufficient for the entire selection process, but this varies depending on the required concentration in the selections and the number of sorting rounds.

2. Dissolve 1 mg of biotin from the EZ-Link Sulfo-NHS-LC-LC-Biotin No-Weigh Format kit (EZ-Link Sulfo-NHS-LC-LC-Biotin No-Weigh Format) in 150 μL ddH_2_0.

***Note:*** Make sure that biotin is completely dissolved. Use the biotin directly after dissolving, do not use it after extended storage.

3. Add the biotin reagent in 5x molar excess to the protein. Gently mix by pipetting up and down and incubate for 30 min at 20°C.

***Note:*** Refer to the manufacturer’s protocol for calculations (EZ-Link Sulfo-NHS-LC-LC-Biotin No-Weigh Format). Do not use higher excess of biotin to avoid heavily biotinylated proteins, which might negatively affect binder selections. This ensures optimal binding specificity and prevents interference in selection processes.

4. Add 1 M TRIS stock buffer to achieve final concentration of 3 mM to saturate excess free reactive NHS-biotin molecules.
5. Remove excess biotin with PD-10 desalting columns according to the manufacturer’s instructions (PD-10 desalting columns) using PBS as equilibration and elution buffer.
6. Measure the concentration and store aliquots of the biotinylated antigen at −80°C.

***Optional:*** Excess biotin can also be removed by dialysis or size-exclusion chromatography. Refer to the manufacturer’s protocol for recommendations (PD-10 desalting columns).

## Key resources table

**Table:** 

REAGENT or RESOURCE	SOURCE	IDENTIFIER
**Antibodies**		

Penta-His Alexa Fluor 647 conjugate, 1:40	QIAGEN	Cat# 35370, RRID:AB_3083468
Penta-His Alexa Fluor 488 conjugate, 1:40	QIAGEN	Cat# 35310, RRID:AB_3083465
Mouse anti-c-myc (clone 9E10) , 1:50, 1:100, and 1:500	Thermo Fisher Scientific	Cat# 13-2500, RRID:AB_2533008
Goat anti-mouse IgG Alexa Fluor 488, 1:100 and 1:200	Thermo Fisher Scientific	Cat# A-11001, RRID:AB_2534069
Anti-HA.11-AF488 (clone 16b12) , 1:500	BioLegend	Cat# 901509, RRID:AB_2565072
Anti-HA.11-AF647 (clone 16b12) , 1:500	BioLegend	Cat# 682404, RRID:AB_2566616

**Bacterial and virus strains**		

NEB 10-beta electrocompetent *Escherichia coli*	New England Biolabs	Cat# C3020K

**Chemicals, peptides, and recombinant proteins**		

Animal-free recombinant human EGF	PeproTech	Cat# AF-100-15, Gene ID: 1950
EGFR-1xG4S-Fc	Dobersberger et al.^1^	N/A
Yeast nitrogen base	Thermo Fisher Scientific	Cat# 291940
Bacto casamino acids	BD	Cat# 223120
Streptavidin-AF647, 1:100	Thermo Fisher Scientific	Cat# S32357
dPTP	Jena Bioscience	Cat# NU-1119S
8-oxo-dGTP	Jena Bioscience	Cat# NU-1117S
BamHI-HF	New England Biolabs	Cat# R3136S
NheI-HF	New England Biolabs	Cat# R3131S
SalI-HF	New England Biolabs	Cat# R3138S

**Critical commercial assays**		

EZ-Link Sulfo-NHS-LC-LC-biotin No-Weigh format	Thermo Fisher Scientific	Cat# 21338
Zymoprep Yeast Plasmid Miniprep II kit	Zymo Research	Cat# D2004
Monarch DNA gel extraction kit	New England Biolabs	Cat# T1020L
Frozen-EZ Yeast Transformation II kit	Zymo Research	Cat# T2001
Monarch PCR & DNA cleanup kit	New England Biolabs	Cat# T1030L

**Experimental models: Organisms/strains**		

*S. cerevisiae*: EBY100	ATCC	ATCC MYA-4941

**Oligonucleotides**		

Primer 1: GGCTCTGGTGGAGGCGGTAGCGG AGGCGGAGGGTCGGCTAGC	N/A	In this paper
Primer 2: CTATTACAAGTCCTCTTCAGA AATAAGCTTTTGTTCGGATCC	N/A	In this paper
Sequencing primer: CGTTTGTCAGT AATTGCGGTTCTC	N/A	In this paper

**Recombinant DNA**		

pCTCON2V	GenScript	N/A

**Software and algorithms**		

FlowJo v10.8.1	BD Life Sciences	RRID: SCR_00852
BioRender	BioRender	RRID: SCR_01836

**Other**		

Yeast-display library rcSso7d-11	Traxlmayr et al.^8^	N/A
Yeast-display library rcSso7d-18	Traxlmayr et al.^8^	N/A
SH800S cell sorter	Sony Biotechnology	N/A
FACSAria Fusion cell sorter	BD Biosciences	N/A
CytoFLEX S	Beckman Coulter	N/A
Gene Pulser Xcell electroporation system	Bio-Rad	Cat# 1652661
DynaMag-2 magnetic stand	Invitrogen	Cat# 12321D
Rotating wheel	N/A	N/A
Thermoshaker (preferred) or heat block	N/A	N/A
Dynabeads biotin binder	Thermo Fisher Scientific	Cat# 11047
0.22 μm filter (Steritop)	Millipore	Cat# SCGPS02RE
Cooled micro centrifuge for 1.5–2.0 mL tubes	N/A	N/A
Cooled tabletop centrifuge for 50 mL tubes	N/A	N/A

## Materials and equipment

**Table:** PBSA

Reagent	Final concentration	Amount
Bovine serum albumin	1 g/L	1 g
10x PBS	1×	100 mL
ddH_2_O	N/A	900 mL
**Total**	**N/A**	**1,000 mL**

Filter-sterilize through a 0.22 μm filter and store at 4°C. The buffer can be used for at least 3 months.

**Table:** SD-CAA

Reagent	Final concentration	Amount
D-Glucose	20 g/L	20 g
Yeast nitrogen base	6.7 g/L	6.7 g
Casamino acids	5 g/L	5 g
Tri-sodium citrate dihydrate	10.83 g/L	10.83 g
Citric acid monohydrate	7.4 g/L	7.4 g
ddH_2_O	N/A	Fill up to 1,000 mL
**Total**	**N/A**	**1,000 mL**

Filter-sterilize through a 0.22 μm filter and store at 20°C–25°C. The buffer can be used for at least 3 months.

**Table:** SG-CAA

Reagent	Final concentration	Amount
D-Glucose	2 g/L	2 g
Galactose	20 g/L	20 g
Yeast nitrogen base	6.7 g/L	6.7 g
Casamino acids	5 g/L	5 g
di-sodium hydrogen phosphate heptahydrate	10.20 g/L	10.20 g
Sodium dihydrogen phosphate monohydrate	8.56 g/L	8.56 g
ddH_2_O	N/A	Fill up to 1,000 mL
**Total**	**N/A**	**1,000 mL**

Filter-sterilize through a 0.22 μm filter and store at 20°C–25°C. The buffer can be used for at least 3 months.

**Table:** SD-CAA plates

Reagent	Final concentration	Amount
di-sodium hydrogen phosphate heptahydrate	10.20 g/L	5.10 g
Sodium dihydrogen phosphate monohydrate	7.44 g/L	3.72 g
Agar	15 g/L	7.5 g
Sorbitol	182 g/L	91 g
D-Glucose	20 g/L	10 g
Yeast nitrogen base	6.7 g/L	3.35 g
Casamino acids	5.0 g/L	2.5 g
ddH_2_O	N/A	Fill up to 500 mL
**Total**	**N/A**	**500 mL**

Store at 4°C. The plates can be used for at least 3 months.

***Note:*** Dissolve sorbitol, agar and buffer salts in ddH_2_O and fill up to 450 mL and autoclave. Dissolve glucose, casamino acids and yeast nitrogen base in ddH_2_O and fill up to 50 mL. Filter-sterilize through a 0.22 μm filter. Add to 55°C cooled autoclaved agar before pouring plates.

**Table:** YPD agar

Reagent	Final concentration	Amount
Peptone	20 g/L	10 g
Yeast extract	10 g/L	5 g
Agar	15 g/L	7.5 g
Glucose solution	20 g/L	50 mL
ddH_2_O	N/A	450 mL
**Total**	**N/A**	**500 mL**

Store at 4°C. The plates can be used for at least 1 month.

***Note:*** Dissolve peptone, yeast extract and agar in ddH2O and fill up to 450 mL. Autoclave and add 50 mL of a sterile glucose stock solution (200 g/L) to 55°C cooled agar before pouring plates.

**Table:** YPD medium

Reagent	Final concentration	Amount
Peptone	20 g/L	10 g
Yeast extract	10 g/L	5 g
Glucose solution	20 g/L	50 mL
ddH_2_O	N/A	450 mL
**Total**	**N/A**	**500 mL**

Store at 4°C. The medium can be used for at least 1 month.

***Note:*** Dissolve peptone and yeast extract in ddH2O and fill up to 450 mL. Autoclave and add 50 mL of a sterile glucose stock solution (200 g/L).

**Table:** TRIS stock buffer

Reagent	Final concentration	Amount
TRIS	1 M	6.06 g
ddH_2_O	N/A	Fill up to 50 mL
**Total**	**N/A**	**50 mL**

Adjust to pH 7.2. The buffer can be used for at least 6 months.

**Table:** PenStrep 100× stock

Reagent	Final concentration	Amount
Penicillin G sodium salt	10 000 U/mL	0.624 g
Streptomycin sulfate	10 000 μg/mL	1 g
ddH_2_O	N/A	Fill up to 100 mL
**Total**	**N/A**	**100 mL**

Filter-sterilize through a 0.22 μm filter, aliquot and store at −20°C. The stock solution is stable at −20°C for approximately 1 year.

**Table:** PBSA-EGF

Reagent	Final concentration	Amount
PBSA	1x	50 mL
EGF	20 nM	50 μL of 20 μM stock
**Total**	**N/A**	**50 mL**

Prepare freshly and use directly.

**Table:** Lithium acetate buffer

Reagent	Final concentration	Amount
Lithium acetate dihydrate	1 M	10.2 g
ddH_2_O	N/A	Fill up to 100 mL
**Total**	**N/A**	**100 mL**

Filter-sterilize through a 0.22 μm filter. The solution can be used for at least 6 months.

***Note:*** Prepare a 1:10 dilution as a working stock to achieve a final concentration of 100 mM.

## Step-by-step method details

### First round of bead selection

**Timing: 3 days**

This section describes the selection of binding domains from a naïve yeast surface display library through magnetic bead-based selection. For a naïve library, the selection process is typically started with magnetic bead selections (Figure 1A). This enables to select a higher number of yeast cells, which is important for large yeast libraries. Moreover, multivalent binding to the antigen immobilized on the beads helps to enrich low affinity binders,^17^ whose affinities can subsequently be improved via affinity maturation.

1. Thaw the naïve yeast library.
  a. Thaw aliquots of yeast surface display libraries (rcSso7d-11 and rcSso7d-18) at 20°C and quickly transfer them into conical tubes with 30 mL SD-CAA medium.
  b. Spread 100 μL of 10^–5^, 10^–6^ and 10^–7^ dilutions on SD-CAA agar plates.
  c. Incubate plates at 30°C for 2–3 days before counting colonies and determining the viable cell number after thawing.
  d. Use the remaining culture of each library to inoculate 1 L SD-CAA medium in appropriate shake flasks and incubate the cultures (30°C, 180 rpm, >16 h).

***Note:*** 1 L SD-CAA medium can be split up in e.g. two 2 L shake flasks.

***Note:*** Ensure that the viable cell number exceeds the library diversity at least 10x after thawing. The diversities of the yeast libraries rcSso7d-11 and rcSso7d-18 are approximately 1.4×10^9^ each.^8^ Adapt the culture volumes in all steps of this protocol accordingly to cover library diversities by at least 10x.

2. Passage and induce the yeast library.
  a. Measure the optical density at 600 nm (OD_600_) of the overnight cultures and dilute cells to reach a density of 1.4×10^7^ cells/mL in 1 L SD-CAA. This corresponds to a total number of 1.4×10^10^ cells for each library, thus covering the library diversity 10-fold.
    ***Note:*** An OD_600_ of 1 corresponds to 1×10^7^ cells/mL.
  b. Incubate the cultures (30°C, 180 rpm) in 2 L shake flasks for 4–5 h until the mid-log phase is reached (OD_600_ of ∼4).
  c. Centrifuge sufficient cells to cover 10x the diversity of each library (2000 *g*, 5 min, 20°C).
  d. Discard the supernatant and resuspend the pellet in 1 L SG-CAA in 2 L shake flasks to induce display of the protein variants.
  e. Incubate the cultures (20°C, 180 rpm, >16 h).
    ***Note:*** It is best to directly use cells after induction. Induced cells can be stored at 4°C for several days but should only be used for analysis and not for selections.
3. Prepare the antigen-beads.
  a. Prepare two tubes of 100 μL Dynabeads Biotin Binder and 900 μL PBSA in 2 mL microcentrifugation tubes. 1 tube contains beads for 10 selections.
    ***Note:*** This protocol uses 10 μL Dynabeads Biotin Binder for 1 selection (in 1 final tube) containing 1.2×10^9^ cells.
  b. Mix well by inverting the tubes 3–4 times.
  c. Place the tubes on the magnetic rack for 2 min.
    **CRITICAL:** Whenever placing a tube on the magnetic rack, always use a pipet to transfer the liquid trapped in the lid into the tube.
  d. Remove the PBSA and resuspend beads in 1 mL PBSA.
    **CRITICAL:** Carefully move the pipet tip along the wall of the tube opposite to the beads to not remove any beads. Beads should be visible in the tube.
  e. Repeat the separation on the magnetic rack and resuspend washed beads in 1 mL PBSA with 100 pmol biotinylated EGFR-Fc and 2000 pmol EGF.
    ***Note:*** This corresponds to an antigen density of 10 pmol per tube containing 1.2×10^9^ cells in the final selection. Antigen densities of 6–33 pmol per tube are recommended.^2^^,^^13^ For selection against ligand-receptor complexes, it is necessary to ensure high molar excess of the ligand. This avoids selection against the receptor without the bound ligand.
    ***Optional:*** Centrifuge the thawed antigen (9000 *g*, 10 min, 4°C) to remove large aggregates and determine the protein concentration. If the antigen is known to be stable over freeze-thaw cycles, this step can be skipped.
  f. Incubate for 2 h at 4°C on a tube rotator.
    ***Note:*** During the incubation of antigen-beads the yeast cell cultures can be prepared as described in step 4.
  g. After incubation, place the tubes containing antigen-beads on a magnetic rack for 2 min.
  h. Diligently remove the antigen-containing buffer from the beads to discard excess unbound antigen.
  i. For washing, resuspend antigen-beads in 1 mL PBSA containing 20 nM EGF (PBSA-EGF), repeat the separation on the magnetic rack and discard PBSA-EGF.
    ***Note:*** Increasing the ligand concentration in wash buffers may help to keep the receptor in a ligand-bound state.
  j. Repeat this washing step with 1 mL of PBSA-EGF.
  k. Resuspend the washed antigen-beads in 550 μL PBSA-EGF per tube and pool tubes.
4. Prepare the yeast cells.
  ***Note:*** Preparation of cells can be performed during the 2 h incubation of antigen-beads. Washed and aliquoted cells can be kept at 4°C or on ice until bead incubation with the antigen is finished.
  a. Measure OD_600_ of the induced yeast cell culture and centrifuge 10x the diversity of each induced library (2000 g, 10 min, 4°C) in 50 mL conical tubes.
  b. Discard the supernatant and resuspend the cells in 1 mL PBSA. Pool the cells of each library separately.
  c. Flush residual conical tube with 1 mL PBSA (for all) and add it to the pooled libraries.
  d. Take a 5 μL sample of each library, dilute it with 500 μL PBSA and store it at 4°C.
    ***Note:*** This sample is used for later flow cytometric analysis to determine the display level of the yeast cells as described in step 8.
  e. Pool both libraries in a conical tube and fill up to 20 mL with PBSA.
  f. Centrifuge cells (2000 *g*, 10 min, 4°C), discard the supernatant and subsequently resuspend cells in 20 mL PBSA.
  g. Split cell suspensions into 1 mL aliquots in 2 mL microcentrifugation tubes corresponding to 1.2×10^9^ cells per tube.
  h. Centrifuge cells (2000 *g*, 3 min, 4°C) and resuspend cell pellets in 850 μL PBSA-EGF to reach a final volume of 950 μL per tube.
5. Incubate the library with the antigen-beads (Figure 1B).
  a. Add 50 μL of washed antigen-beads to each tube of cells.
  b. Incubate tubes at 4°C for 2 h on a tube rotator.

**CRITICAL:** All following steps are performed on ice with chilled reagents or in a 4°C environment.

6. Wash the bead-bound cells (Figure 1B).
  a. Place the tubes containing cells and the antigen-beads on the magnetic rack for 2 min.
  b. Discard unbound cells.
    **CRITICAL:** The magnetic beads should be visible after removing unbound cells. Do not forget to remove the liquid trapped in the lid.
  c. Resuspend bead-bound cells in 1 mL PBSA-EGF.
  d. Invert the tubes 3–4 times.
  e. Place the tubes on the magnetic rack for 2 min.
  f. Discard the PBSA-EGF and resuspend the bead-bound cells in 1 mL SD-CAA.
7. Cultivate the bead-bound cells and determine the maximum diversity of the sub-library.
  a. Pool the resuspended cells in a final volume of 70 mL SD-CAA with 700 μL PenStrep.
  b. Prepare 1 mL of a 1:20 and 1:200 dilution in SD-CAA.
    i. Spread 50 μL of each dilution on SD-CAA plates and incubate them at 30°C for 3 days.
    ii. Use the colony count to approximate the maximum diversity of the selected library.
      ***Note:*** Since many of these cells will contain identical plasmids and protein variants (because the original library diversity was covered 10-fold), this new diversity should be considered as a maximum value and the real diversity will be lower.
  c. Incubate the remaining yeast culture at 30°C and shaking for >16 h.
8. Determine the display level of the induced libraries.
  ***Note:*** Samples for flow cytometric analysis of the display level do not need to be measured on the day of bead selection. They can be stored at 4°C for up to one week before analysis.
  a. For each sample, pipet 2×10^6^ cells into a well of a V-bottom 96-well plate.
  b. Add 150 μL PBSA to each sample well and centrifuge cells (2000 *g*, 3 min, 4°C).
  c. Discard supernatant.
    ***Note:*** Discard supernatant of 96-well plates always by inverting the plate with one rapid movement.
  d. Stain cells in 50 μL PBSA with 1:500 diluted mouse anti-c-myc antibody for 20 min at 4°C while shaking.
  e. Centrifuge the cells (2000 *g*, 3 min, 4°C), discard the supernatant and wash with 200 μL PBSA.
  f. Centrifuge the cells (2000 *g*, 3 min, 4°C) and discard the supernatant.
  g. Stain cells in 50 μL PBSA with 1:200 diluted goat anti-mouse IgG AF488 antibody for 20 min at 4°C on an orbital shaker.
  h. Wash the plate as described in step 8c-e.
  i. Resuspend cells in 100 μL PBSA just before the measurement with a flow cytometer.
    **Pause point:** SD-CAA cultures can be stored at 4°C for up to one month. Additionally, the libraries can be frozen and stored in SD-CAA containing 15% glycerol at −80°C for an extended period after removing the beads as described in step 9 of this protocol. After storage at −80°C, the library can be thawed and induced at a later time point.

### Second round of bead selection

**Timing: 2 days**

Subsequent rounds of bead-based selections should be performed to further enrich binders and reduce the library diversity for later flow cytometric sorts (Figure 1A). These rounds should also contain negative bead selection steps to deplete binders against bare beads. Before these negative selections, it is important to remove residual antigen-coated beads remaining from the first positive selection, since these beads would cause depletion of antigen-specific binders in the negative selections.

9. Remove the beads from the previously selected library and induce the library.
  a. Measure OD_600_ and centrifuge at least 10x the diversity of the library (2000 *g*, 3 min, 20°C) in 50 mL conical tubes.
  b. Resuspend cells in 1 mL SD-CAA and transfer to a 2 mL microcentrifuge tube.
  c. Place tube on a magnetic rack for 2 min.
  d. Transfer the unbound cells to a clean 2 mL microcentrifuge tube.
    ***Note:*** Do not discard the unbound cells. Residual antigen-beads must be removed prior to negative selection against bare beads.
  e. Repeat the separation process on the magnetic rack.
  f. Dilute the unbound cells in SD-CAA to an OD_600_ of 1 and incubate (30°C, 180 rpm) for 4–5 h until an OD_600_ of ∼4 is reached.
  g. Centrifuge 10x the diversity of the library (2000 *g*, 3 min, 20°C–25°C ) and resuspend cells in SG-CAA to an OD_600_ of 1.
  h. Incubate the cultures (20°C, 180 rpm, >16 h).
10. Prepare the bare beads.
  a. Add 30 μL Dynabeads Biotin Binder to 970 μL PBSA in a 2 mL tube. Mix well by inverting the tube 3–4 times.
  b. Place the tube on the magnetic rack for 2 min.
  c. Remove the PBSA and resuspend beads in 1 mL PBSA.
  d. Repeat separation on magnetic rack and resuspend washed beads in 150 μL PBSA.
11. Prepare the yeast cells.
  a. Centrifuge 10x the diversity of the library (2000 *g*, 3 min, 4°C) in a 50 mL conical tube.
  b. Remove medium and resuspend cells in 1 mL PBSA in a 2 mL microcentrifuge tube.
  c. Centrifuge cells (2000 *g*, 3 min, 4°C) and resuspend in 1 mL PBSA.
  d. Centrifuge cells (2000 *g*, 3 min, 4°C) and resuspend in PBSA to reach a final volume of 950 μL.
  e. Take a 5 μL sample, dilute it with 500 μL PBSA and store it at 4°C for later flow cytometric analysis of the display level.
12. Perform three negative selections with bare beads (Figure 1A).
  a. Add 50 μL of washed bare beads to the 2 mL microfuge tube containing the washed cells.
  b. Incubate the tube at 4°C for 1.5 h on a tube rotator (1^st^ negative selection).
    **CRITICAL:** All following steps are performed with chilled reagents and on ice or in a 4°C environment.
    ***Note:*** During incubation of the 1^st^ or 2^nd^ negative selection, antigen-beads can be prepared as described in step 13.
  c. Place the tube on the magnetic rack for 2 min.
  d. Pipet the non-binding cells into a fresh 2 mL tube.
    **CRITICAL:** Do not discard non-binding cells. Do not discard the bare beads.
  e. Add 50 μL of washed bare beads to the tube containing the non-binding cells and incubate at 4°C for 1.5 h on a tube rotator (2^nd^ negative selection).
  f. Wash and store the bare beads from the 1^st^ negative selection.
    i. Add 1 mL PBSA to bare-beads from 1^st^ negative selection after removing non-binding cells.
    ii. Mix by inverting the tube and place the tube on the magnetic rack for 2 min.
    iii. Discard the PBSA, resuspend beads in 1 mL PBSA and repeat separation on magnetic rack.
    iv. Discard the PBSA, resuspend beads in 30 mL SD-CAA and add 300 μL PenStrep.
    v. Prepare a 1:5 and 1:10 dilution in SD-CAA and spread 20 μL of each dilution on SD-CAA plates.
    vi. Incubate plates at 30°C for 2–3 days before counting colonies.
      ***Note:*** The number of cells from the 1^st^ negative selection compared to those from the positive selection indicates the enrichment of antigen-specific binders.
  g. Place tubes from 2^nd^ negative selection on the magnetic rack for 2 min.
  h. Pipet the non-binding cells into a fresh 2 mL microcentrifuge tube.
  i. Add 50 μL of washed bare beads to the tube containing the non-binding cells.
  j. Incubate at 4°C for 1.5 h on a tube rotator (3^rd^ negative selection).
  k. Discard the beads from the 2^nd^ negative selection.
  l. Place tubes from 3^rd^ negative selection on the magnetic rack for 2 min.
  m. Transfer non-binding cells to a clean 2 mL microcentrifuge tube.
  n. Discard the beads from the 3^rd^ negative selection.
13. Prepare the antigen-beads.
  ***Note:*** Preparation of antigen beads can be done during the incubation of the first or second negative selection.
  a. Add 10 μL Dynabeads Biotin Binder to 990 μL PBSA in a 2 mL tube. Mix well by inverting the tube 3–4 times.
  b. Place the tube on the magnetic rack for 2 min.
  c. Remove the PBSA and resuspend beads in 1 mL PBSA.
  d. Repeat separation on magnetic rack and resuspend washed beads in 100 μL buffer containing 10 pmol biotinylated EGFR-Fc and 200 pmol EGF.
  e. Incubate for 2 h at 4°C on a tube rotator.
  f. After incubation, add 800 μL PBSA-EGF and place the tube containing antigen-beads on a magnetic rack for 2 min.
  g. Discard the antigen-containing buffer.
  h. For washing, resuspend antigen-beads in 1 mL PBSA-EGF, repeat the separation on the magnetic rack and discard the PBSA-EGF.
  i. Repeat this washing step.
  j. Resuspend antigen-beads in 60 μL PBSA-EGF and store on ice until further use.
14. Incubate the library with the antigen-beads.
  a. Add 50 μL of antigen-beads to the tube containing non-binding cells from the 3^rd^ negative selection.
  b. Incubate the tube at 4°C for 2 h on a tube rotator.
15. Wash and cultivate bead-bound cells.
  a. Place the tube containing cells and the antigen-beads on the magnetic rack for 2 min.
  b. Discard unbound cells and resuspend bead-bound cells in 1 mL PBSA-EGF.
  c. Invert the tube, place the tubes on the magnetic rack for 2 min and discard PBSA-EGF with unbound cells.
  d. Repeat the washing step and separation process.
  e. Discard the PBSA-EGF and resuspend the beads in 30 mL SD-CAA with 300 μL PenStrep.
  f. Prepare a 1:10 dilution in SD-CAA and spread 20 μL on SD-CAA plates. Incubate plates at 30°C for 2–3 days.
    ***Note:*** Use the colony count to approximate the diversity of the positively selected library and compare to the diversity of the library of the 1^st^ negative selection. Again, this new diversity should be considered as a maximum value and the real diversity will be lower, since individual clones are expected to be present multiple times due to oversampling of the library diversity.
  g. Incubate the remaining liquid culture (30°C, 180 rpm, >16 h).
  h. Measure OD_600_ of the overnight culture and separate library from beads as described in step 9, before further using the library.
    ***Note:*** Culture volumes should be adapted to the new library diversity.
16. Measure display level of the induced library used for the second round of bead selection (sample taken in step 11e) as described in step 8.

**Pause point:** Libraries can be stored at 4°C up to one month or they can be frozen and stored in SD-CAA containing 15% glycerol at −80°C for an extended period.

### Isolation of plasmids and random mutagenesis to increase diversity

**Timing: 3 days**

The plasmids of the enriched binders are isolated to insert random mutations by error-prone PCR (epPCR),^2^^,^^13^ thereby again increasing the diversity of the library. In this way, new binder variants with enhanced binding affinities are introduced into the library.

17. Isolate the plasmids according to the manufacturer’s instructions with the modification of adding 8 μL of Zymolyase and incubating for 3 h (Zymoprep Yeast Plasmid Miniprep II Kit).

***Note:*** The isolated plasmids serve as templates for the following error-prone PCR. Isolated plasmids can be stored at −20°C until further usage.

18. Perform an error-prone PCR as described by Chen et al.^13^ to increase diversity of the library with the conditions described below (Tables 1 and 2).

**Table 1:** Error-prone PCR reaction mix (50 μL)

Reagent	Amount
DNA template	6 μL
Taq Polymerase	0.5 μL
Primer 1 (10 μM)	2.5 μL
Primer 2 (10 μM)	2.5 μL
10x Thermopol buffer	5 μL
dNTPs (10 mM)	1 μL
8-oxo-dGTP (100 μM)	1 μL
dPTP (100 μM)	1 μL
ddH_2_O	30 μL

**Table 2:** Error-prone PCR cycling conditions

Steps	Temperature	Time	Cycles
Initial denaturation	94°C	3 min	1
Denaturation	94°C	45 s	18 cycles
Annealing	60°C	30 s	
Extension	72°C	1 min	
Final extension	72°C	10 min	1
Hold	4°C	forever
19. Isolate and purify the amplified inserts from a preparative agarose gel.
  a. Prepare a preparative agarose gel (2%).
  b. Mix PCR reaction with 10 μL of 6x loading dye.
  c. Load 8 μL of GeneRuler 1 kB DNA ladder (1:5 diluted).
  d. Run the gel at 120 V for 45 min.
  e. Perform a gel extraction of the band at the expected size according to the manufacturer’s protocol (Monarch DNA Gel Extraction Kit).
  f. Determine the concentration of the purified DNA.
20. Amplify the randomly mutated and purified insert DNA with the conditions listed below (Tables 3 and 4).

**Table 3:** PCR reaction master mix (2 × 100 μL)

Reagent	Amount
DNA template	40 ng
Q5 Polymerase	1 μL
Primer 1 (10 μM)	10 μL
Primer 2 (10 μM)	10 μL
5x Q5 buffer	20 μL
dNTPs (10 mM)	2 μL
ddH_2_O	Up to 100 μL

**Table 4:** PCR cycling conditions

Steps	Temperature	Time	Cycles
Initial denaturation	98°C	3 min	1
Denaturation	98°C	45 s	29 cycles
Annealing	60°C	30 s	
Extension	72°C	1 min	
Final extension	72°C	10 min	1
Hold	4°C	forever
21. Prepare an analytical agarose gel (2%) to verify amplified inserts.
  a. Use 5 μL PCR-product and add 1 μL of 6x loading dye.
  b. Load 8 μL of GeneRuler 1 kB DNA ladder (1:5 diluted).
  c. Run the gel at 120 V for 45 min.

***Note:*** This step confirms successful amplification and correct size of the insert.

22. Purify the PCR product by ethanol precipitation.
  a. Add 10% volume of 3 M sodium acetate, pH 5.2 (starting volume 200 μL).
  b. Add twice the volume of 100% ethanol.
  c. Incubate at 20°C–25°C for 2 min.
  d. Centrifuge (18 000 *g*, 5 min) and remove the supernatant.
    ***Note:*** A pellet should be visible.
  e. Add 500 μL of 70% ethanol and mix briefly.
  f. Centrifuge (18 000 *g*, 5 min) ) and remove the supernatant.
  g. Add 500 μL of 100% ethanol and mix briefly.
  h. Centrifuge (18 000 *g*, 5 min).
  i. Remove supernatant.
  j. Let the pellet dry with the lid open until there is no liquid left.
    ***Note:*** This process takes usually a few hours.
  k. Dissolve the dried DNA pellet in 10 μL sterile nuclease-free water and store at −20°C.
    ***Note:*** The sterile nuclease-free water must be of high purity to prevent DNA degradation and to avoid any negative impact on the following electroporation.
23. Linearize and digest the pCTCON2V vector.
  ***Note:*** The vector pCTCON2V is cleaved at three specific restriction sites to ensure that upon linearization, it can solely circularize in the presence of the insert.
  a. Linearize the vector with SalI-HF at 37°C for 24 h (Table 5).
    ***Note:*** The digest can be scaled up or down.

**Table 5:** Mix for vector linearization

Reagent	Amount
pCTCON2V template	20 μg
CutSmart buffer (10x)	5 μL
SalI-HF (20 U/μL)	3 μL
ddH_2_O	Up to 50 μL
  b. Perform a double digest of the linearized vector with restriction enzymes NheI-HF and BamHI-HF at 37°C for 24 h (Table 6).
    ***Note:*** Extended digestion is recommended to reduce the background of undigested vector. Both enzymes and buffer can be added directly to the reaction tube for vector linearization.

**Table 6:** Mix for double digest of linearized vector

Reagent	Amount
pCTCON2V (SalI digested)	50 μL
CutSmart buffer (10x)	3.75 μL
BamHI-HF (20 U/μL)	1.5 μL
NheI-HF (20 U/μL)	1.5 μL
ddH_2_O	Up to 87.5 μL
  c. Precipitate and analyze the digested vector as described in steps 21 and 22.
24. Electroporate yeast with the amplified randomized inserts and digested vector.
  ***Note:*** Yeast cells will insert the randomized inserts amplified with overhangs to the digested linearized vector by homologous recombination. For that purpose, the insert and the linearized vector should contain homologous overhangs of 30–50 bp on both ends.^2^
  ***Note:*** As controls, electroporate yeast without vector and insert (“cells only”) and without insert (“vector only”).
  a. Strike out *S. cerevisiae* EBY100 on YPD agar plates and incubate for 2–3 days.
  b. Inoculate 20 mL freshly prepared YPD medium with a single colony of EBY100.Incubate while shaking for >16 h at 30°C.
  c. Dilute EBY100 cells to an OD_600_ of 0.2.
    ***Note:*** The culture volume depends on the number of samples to be electroporated. 25 mL are required for one electroporation.
  d. Keep shaking at 30°C until OD_600_ of 1.3 to 1.5 is reached (4–6 h). Troubleshooting 2.
  e. Centrifuge 50 mL of culture (2000 *g*, 3 min) in a 50 mL conical tube and discard supernatant.
  f. Resuspend cells in 25 mL (half of the centrifuged culture volume) of 100 mM lithium acetate.
  g. Add 250 μL of freshly prepared and sterile filtered 1 M DTT solution to reach a final concentration 10 mM.
  h. Incubate cells (30°C, 180 rpm, 10 min).
  i. Centrifuge cells (2000 *g*, 3 min), discard supernatant and place on ice immediately.
    **CRITICAL:** All the following steps will be performed on ice and with chilled reagents and cuvettes.
  j. Resuspend cells in 25 mL (half of the centrifuged culture volume) ice-cold sterile H_2_O.
  k. Centrifuge (2000 *g*, 3 min) and discard the supernatant.
  l. Resuspend cells in 350–400 μL ice-cold sterile H_2_O, which will roughly yield 500 μL total volume.
  m. Electroporate cells.
  n. Add 4 μg of linearized pCTCON2V vector to the tube containing the amplified DNA inserts (dissolved in 10 μL sterile nuclease-free water (step 22j).
    i. Add 250 μL cells to the vector-insert mix.
    ii. Transfer the mix to pre-chilled 2 mm electroporation cuvettes.
    iii. Electroporate cells with a Bio-Rad Gene Pulser Xcell using the square wave function (single pulse, 500 V, 15 ms pulse duration).
  o. Immediately transfer cells to a tube containing 0.5 mL pre-warmed YPD medium.
    i. Rinse the cuvette with 0.5 mL YPD and add to the microcentrifugation tube.
    ii. Keep cells on a heat block at 30°C until all samples and controls are electroporated.
  p. Transfer electroporated cells to a 15 mL tube and incubate at 30°C without shaking for 1 h.
    i. Perform serial dilutions of the cell suspension of the new libraries in SD-CAA and plate on SD-CAA plates to determine the number of transformants (which yields the new library diversity).
      ***Note:*** The new library diversity is usually in the range of 1 × 10^6^–1×10^8^.
    ii. Centrifuge the liquid cultures for the “cells only” and “vector only” controls (2000 *g*, 3 min), resuspended in 1 mL SD-CAA and plate 100 μL of 10^–1^, 10^–2^ and 10^–3^ dilutions on SD-CAA plates. Troubleshooting 3.
    iii. Incubate the plates at 30°C for 2–3 days.
  q. Centrifuge the remaining cells (2000 *g*, 3 min), resuspend in 200 mL SD-CAA medium and grow at 30°C while shaking for >16 h.
    ***Note:*** The new library can either be induced in SG-CAA directly on the following day (covering a 10x diversity) or passaged in SD-CAA, which will decrease the fraction of untransformed cells.
    ***Note:*** Depending on the library diversity and binding behavior, the library can be selected via flow cytometry instead of magnetic bead-based selections after error prone PCR. While flow cytometric sorting exhibits lower throughput, it presents the advantage of enabling the selection of specific populations, such as those with low and/or high affinity. Library sizes of 10^7^ clones are feasible to sort via flow cytometry. For the selection described in Dobersberger et al.^1^ an additional round of bead sorting was performed.

### Positive flow cytometric selection

**Timing: 3 days**

**Figure 2:**
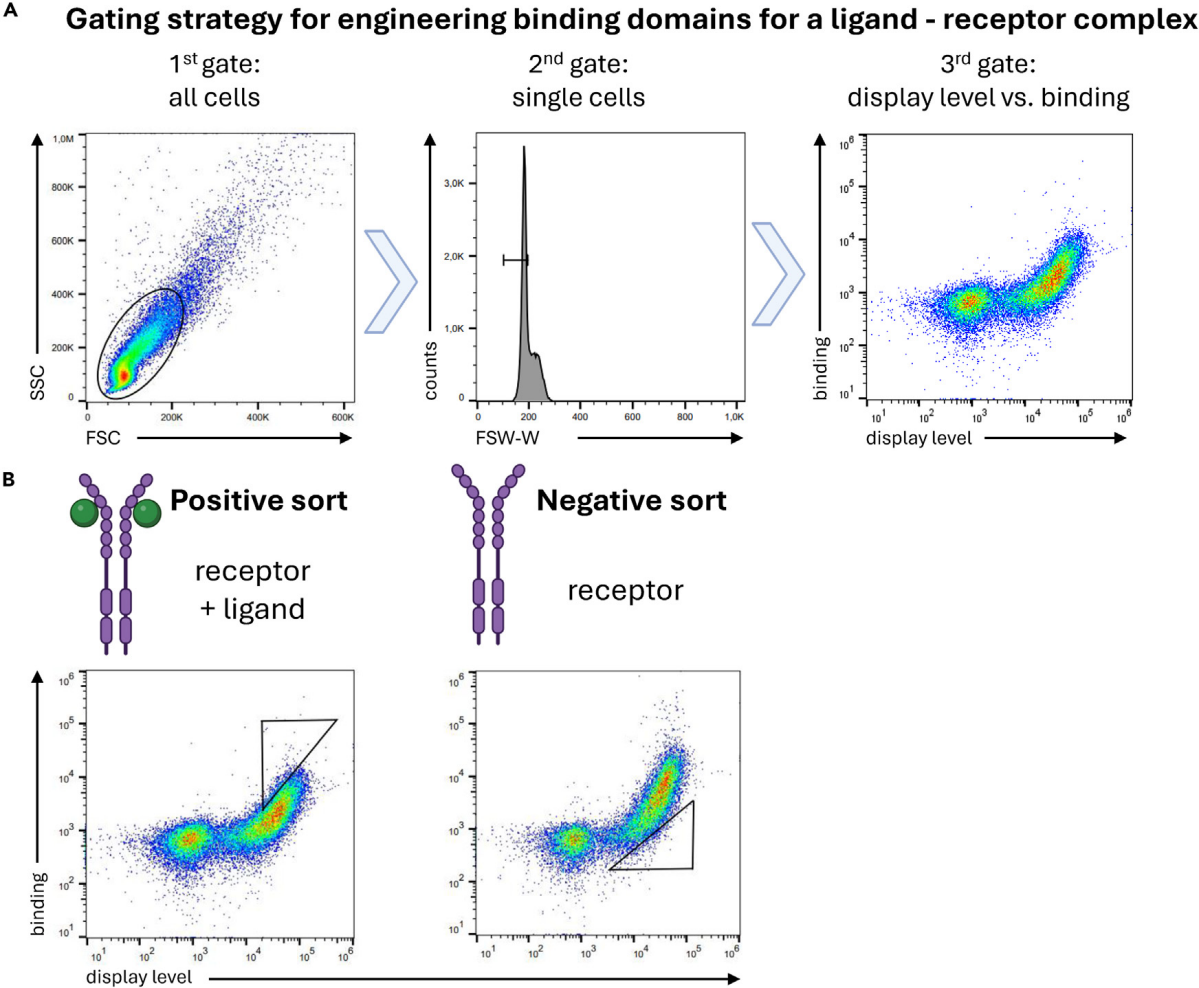
Gating strategy for engineering binding domains (A) The applied gating strategy for selection of binding domains specific for a ligand-receptor complex. The first gate is showing the forward scatter (FSC) versus the side scatter (SSC). The second gate is drawn to select single yeast cells and remove aggregates (FSC-width, FSC-W). The third gate shows the display level (staining of the respective tag, here: c-myc tag) on the x-axis and the binding of the ligand–receptor complex on the y-axis. (B) Representative gating strategies for positive and negative selections, in which both the ligand and the receptor or only the receptor are used for staining, respectively. Data in (A) and (B) were analyzed with FlowJo.

Positive flow cytometric selections are performed to further enrich binders recognizing the ligand-receptor complex in pre-selected libraries. In those sorts, the libraries can be quantitatively screened for binding to ligand-bound EGFR. For that purpose, antibodies are used for detection of the display of the binder as well as of binding to EGFR-Fc (Figure 2). Troubleshooting 1.

25. Passage and induce the library as described in steps 1 and 2 in 15 mL medium and make sure to cover 10x diversity of the library (if necessary, increase the culture volume accordingly).
26. Stain the cells.
  a. Centrifuge at least 10x the diversity of the induced library (2000 *g*, 3 min, 4°C) in three 1.5 mL tubes.
  b. Discard the supernatant and wash cells with 1 mL PBSA per tube (2000 g, 3 min, 4°C).
    ***Note:*** All further centrifugation steps are performed similarly.
  c. Centrifuge cells, discard the supernatant and wash cells with 1 mL PBSA.
  d. Centrifuge cells and discard the supernatant.
  e. Resuspend cells in 0.3 mL PBSA (“unstained cells”), in 0.3 mL PBSA with 1:50 diluted mouse anti-c-myc antibody (“expression control”) or 0.3 mL PBSA containing biotinylated EGFR-Fc, 20x molar excess of EGF (compared to the EGFR-Fc concentration) and 1:50 diluted mouse anti-c-myc antibody (“sample”).
    ***Note:*** The required concentration of the EGFR-Fc antigen can be tested in a preliminary experiment by titrating the libraries with the antigen. Troubleshooting 4.
    ***Note:*** The antigen concentration can be gradually reduced in consecutive selection rounds to enrich high-affinity populations. However, it is important that the antigen is in at least tenfold stoichiometric excess compared to the binding scaffolds displayed on yeast. Yeast cells display approximately 5×10^4^ binders per cell. When using low antigen concentrations, adjustments to the volume or cell number may be necessary to maintain an excess of antigen.^18^
  f. Incubate for 1 h at 4°C on a tube rotator.
    ***Note:*** For very low antigen concentrations (low nM or pM range) longer incubation periods may be necessary to achieve equilibrium.^18^
    **CRITICAL:** All following steps are performed with chilled reagents and on ice or in a 4°C environment.
  g. Centrifuge cells, discard the supernatant and wash cells with 1 mL PBSA-EGF.
  h. Centrifuge cells and discard the supernatant.
  i. Resuspend cells in 100 μL PBSA-EGF (“unstained cells”) or in 100 μL PBSA-EGF containing 1:100 diluted goat anti-mouse IgG-AF488 and 1:100 diluted Streptavidin-AF647 (“expression control” and “sample”).
  j. Incubate for 20 min at 4°C on a tube rotator.
  k. Repeat step 26g.
  l. Centrifuge cells, discard the supernatant and keep pellet on ice.
  m. Resuspend cells to a concentration of 5×10^7^ cells/mL in PBSA-EGF just before the sorting process.
  n. Filter them through a cell strainer directly before selection with a cell sorter.
    ***Note:*** In the study by Dobersberger et al.,^1^ the FACS Aria Fusion cell sorter was used for the initial flow cytometric sorting round, followed by the SH800S cell sorter for subsequent rounds.
27. Gating strategy.
  a. Apply a gating strategy as shown in Figure 2A. For positive selections, set a gate to sort the binding population in the presence of the ligand (Figure 2B).
    ***Note:*** In the first positive selection round, all displaying cells, which show binding to the antigen, are collected. In the following rounds, the stringency of the sorting gate can be increased to sort for high affinity clones or different populations. Higher stringency on gates leads to higher enrichment of high-affinity clones, but strongly reduces the diversity of the sorted population. It can be useful to sort different populations with different stringencies at the same time.
  b. Sort cells into a 15 mL conical tube prefilled with 10 mL SD-CAA medium.
    ***Note:*** Depending on the cell sorter and the diversity of the library, different tubes and volumes of SD-CAA medium may be used. Sufficient SD-CAA medium should be supplied to dilute cells and prevent cell lysis due to high concentrations of antifungal-reagents in the sheath fluid of the cell sorter.
  c. Transfer cell suspension to a shake flask and fill up to 30 mL with SD-CAA.
  d. Add 300 μL PenStrep stock solution.
  e. Incubate cells (30°C, 180 rpm, >16 h).
    **Pause point:** Libraries can be stored at 4°C up to one month or they can be frozen and stored in SD-CAA containing 15% glycerol at −80°C for an extended period.

### Negative flow cytometric selection

**Timing: 3 days**

The negative flow cytometric selection is used to deplete binders that interact with the ligand-free state of EGFR. Moreover, these negative selections additionally reduce the frequency of unspecific variants, as well as mutants binding to secondary reagents. This depletion is achieved by incubating the library with EGFR, but in the absence of the ligand EGF, and sorting the non-binding population (Figure 2B). Troubleshooting 1.

28. Passage and induce the library as described in steps 1 and 2 in 10 mL SD-CAA and SG-CAA, respectively.
29. Stain cells.
  a. Centrifuge at least 10x the diversity of the induced library (2000 *g*, 3 min, 4°C) in three 1.5 mL tubes.
  b. Discard the supernatant and resuspend cells in 1 mL PBSA per tube.
    ***Note:*** All further centrifugation steps are performed similarly.
  c. Centrifuge the cells, discard the supernatant and wash cells with 1 mL PBSA.
  d. Centrifuge the cells and discard the supernatant.
  e. Resuspend cells in 0.3 mL PBSA (“unstained cells”), in 0.3 mL PBSA with 1:100 mouse anti-c-myc antibody (“expression control”) or in 0.3 mL PBSA containing biotinylated EGFR and 1:100 mouse anti-c-myc antibody (“sample”).
    ***Note:*** In negative selections, clones that bind to the antigen in the absence of the ligand are depleted and therefore no ligand is added in these stainings.
  f. Incubate for 1 h at 4°C on a tube rotator.
    **CRITICAL:** All following steps are performed with chilled reagents and on ice or in a 4°C environment.
  g. Wash and stain cells further as described in steps 26g-n but use PBSA instead of PBSA-EGF.
    **CRITICAL:** Use PBSA without a ligand present for negative selections.
30. Gating strategy.
  a. For the negative selection, set gates to select the non-binding population as shown in Figure 2B.
  b. Sort cells and cultivate in 5 mL SD-CAA supplemented with 50 μL PenStrep stock solution.
  c. Incubate cells (30°C, 180 rpm, >16 h).

***Note:*** It is expected that after a negative selection, less binders are present in the library. If none remain, the gate might have been too stringent and/or the antigen concentration in the negative selection might have been too high.

**Pause point:** Libraries can be stored at 4°C up to one month or they can be frozen and stored in SD-CAA containing 15% glycerol at −80°C for an extended period.

***Note:*** Continue with alternating positive and negative selections as described within this protocol. Adjust antigen concentrations and gate setting to tune the anticipated binding behavior. This alternating scheme of positive and negative selections should be continued until the population condenses into one defined diagonal, indicating that there is only limited diversity remaining. Alternatively (or in addition), enriched populations from earlier selection rounds may also be sequenced, which typically yields more diverse pools of binders with a wide range of affinities.

### Analysis of single clones on yeast

**Timing: 8 days**

After several rounds of enrichment of a population of binders recognizing EGF-loaded EGFR, the library can be sequenced. Single clones can be selected and further characterized on the surface of yeast.

***Note:*** Due to avidity effects caused by the dimeric architecture of the antigen (EGFR-Fc), a titration on yeast cells is not recommended. If the antigen is a monomer, titrations can be performed to yield the affinity (*K*_D_) as described elsewhere.^18^

31. Isolate the plasmids from the yeast library using the Zymoprep Yeast Plasmid Miniprep II kit (Zymoprep Yeast Plasmid Miniprep II Kit) as described in step 17.
32. Electroporate isolated plasmids into NEB 10-beta electrocompetent *E. coli*.
  **CRITICAL:** It is important to use highly competent cells (i.e. showing high efficacy in the electroporation or chemical transformation), since the concentration of plasmid DNA in yeast minipreps is very low.
  ***Note:*** Any competent cells can be used (e.g. DH5ɑ, TOP10).
  a. Place 1 mm electroporation cuvettes and tubes on ice.
  b. Thaw NEB10-beta electrocompetent cells on ice (10 min) and mix cells by flicking gently.
  c. Transfer 25 μL of the cells to a chilled tube.
  d. Add 1 μL of the isolated plasmids.
  e. Carefully transfer the cell/DNA mix into a chilled cuvette without introducing bubbles and make sure that the cells deposit in the bottom of the cuvette.
  f. Electroporate the cell/DNA mix with a Bio-Rad GenePulser Xcell electroporator using the exponential decay protocol (2.0 kV, 200 Ω, 25 μF).
    ***Note:*** Typical time constants are in the range of 4.8–5.1 ms.
  g. Immediately add 975 μL of NEB outgrowth medium (prewarmed to 37°C) to the cuvette.
    i. Gently pipette up and down twice.
    ii. Transfer to a tube.
    iii. Flush cuvette with medium to recover as many transformed cells as possible.
  h. Incubate at 37°C for 1 h while shaking (300 rpm).
  i. Spread 10 μL, 100 μL and the rest of the cells (after a centrifugation step) onto pre-warmed LB-Amp plates.
  j. Incubate plates for >16 h at 37°C.
33. Pick single clones and determine the DNA sequence with Sanger Sequencing.
34. After sequence analysis select single clones.

***Note:*** Single clones can be selected based on their amino acid composition in the binding interface.

35. Transform the plasmids encoding the selected clones into *S. cerevisiae* EBY100 cells with the Frozen-EZ Yeast Transformation II Kit according to the manufacturer’s instructions (Frozen-EZ Yeast Transformation II Kit).
  a. Spread 50 μL and the residual of the transformation mixture on SD-CAA plates and incubate the plates at 30°C for 2–3 days to allow the transformants to grow.
36. Inoculate 5 mL of SD-CAA medium with a single colony and grow the cells at 30°C and 180 rpm for >16 h.
37. Dilute cells to an OD_600_ of 0.3 in 5 mL SD-CAA and let them grow until the OD_600_ is between 0.8 and 1.
38. Centrifuge cells (2000 *g*, 3 min, 25°C) and discard the supernatant.
39. Resuspend them in 5 mL SG-CAA to induce surface expression.
40. Incubate cells (20°C, 180 rpm, >16 h).
41. Measure OD_600_ and centrifuge the required amount of cells (2000 *g*, 3 min, 4°C) for staining. Discard the supernatant. All further centrifugation steps are performed similarly.

***Note:*** For staining, 3×10^6^ cells per well are used in a 96-plate. More cells can be prepared to help formation of a visible cell pellet.

42. Wash cells with 1 mL of PBSA.
43. Centrifuge cells and discard the supernatant again.
44. Resuspend the cells in an appropriate volume and transfer 3×10^6^ cells in 25 μL into a V-bottom 96-well plate.
45. Add 25 μL containing 30 nM EGFR-Fc either without EGF (“EGFR only”) or pre-incubated with 200 nM EGF (“EGFR+EGF”). This results in a final concentration of 15 nM EGFR-Fc and 100 nM EGF.
46. Incubate for 60 min at 4°C on a plate-shaker.

**CRITICAL:** All following steps are performed with chilled reagents and on ice or in a 4°C environment.

47. Centrifuge cells, discard the supernatant and wash cells with 200 μL PBSA (“EGFR only”) or PBSA-EGF (“EGFR+EGF”).
48. Centrifuge cells and discard the supernatant.
49. Resuspend cells in 25 μL containing 1:40 diluted Penta-His antibody and 1:500 diluted anti-HA, both with different fluorophores, either in PBSA (“EGFR only”) or PBSA-EGF (“EGFR+EGF”).
50. Incubate for 20 min at 4°C on a plate-shaker.
51. Centrifuge cells and discard supernatant.
52. Wash with 200 μL PBSA (“EGFR only”) or PBSA-EGF (“EGFR+EGF”).
53. Centrifuge cells, discard the supernatant and keep pellet on ice.
54. Resuspend cells in 100 μL PBSA or PBSA-EGF just before the flow cytometric measurements. (see Figure 2A for gating).

## Expected outcomes

This protocol describes step-by-step instructions for the engineering of binding domains specifically recognizing a ligand-receptor complex. Magnetic bead-based selections are expected to yield libraries with a range of affinities to the antigen and reduce initial library diversity (e.g., ∼10^9^) to a level that can be processed by flow cytometric selections (i.e., ∼10^7^). Subsequent random mutagenesis through error-prone PCR yields libraries with increased diversity for further screening and affinity maturation. By alternating positive and negative flow cytometric selections, clones with the desired binding properties (e.g., specific binding to a receptor in the presence of a ligand and no or low binding to the same receptor in the absence of that ligand) can be enriched. For examples of successful selections refer to Dobersberger et al.^1^ and Zajc et al.^7^

## Limitations

A limitation of this protocol is that success is highly dependent on the quality of the protein antigen, as well as the stability of the ligand-receptor complex. If the complex dissociates during the selection process (for example due to low affinity between the ligand and the receptor), this may lead to enrichment of binders against the ligand-free state of the antigen during positive selections. Therefore, ligand-receptor complexes with low affinities may require high ligand concentrations during the staining processes to enable complex formation. Another limitation might be the availability of sufficient amounts of antigen and ligand. Since the ligand needs to be present not only during the primary incubations with the receptor but also in the buffer during washing steps and secondary incubations, larger amounts of ligand are required. For flow cytometric sorts, suitable concentrations of both receptor and ligand can be tested in preliminary analytical flow cytometric experiments.

## Troubleshooting

### Problem 1

Binders against biotinylated antigens, fluorophores or streptavidin are enriched (related to the biotinylation of the antigen as well as positive flow cytometric selection and negative flow cytometric selection).

### Potential solution

As the protocol uses biotinylated antigens for bead selections, binders against biotinylated epitopes may be enriched. To avoid enrichment of such binders, selection of a tagged, non-biotinylated antigen may be helpful (e.g., His-tagged antigen). Additionally, during flow cytometric sorts, binders against the secondary staining reagents such as fluorophores or streptavidin may be enriched. Therefore, we recommend to alternate between different detection reagents during flow cytometric sorts. In general, the negative selection step described in this protocol should help deplete undesirable binders against secondary staining reagents.

### Problem 2

EBY100 do not reach OD_600_ of 1.3 within 6 h (related to step 24.d).

### Potential solution

Ensure to use a fresh colony of EBY100 from a YPD plate. Alternatively, prepare also a new bottle of YPD medium.

### Problem 3

High background of “vector only” in step 24.

### Potential solution

Ensure that the linearized pCTCON2V vector is cut properly and for an extended time period as described above. Furthermore, after the double digest (step 23.b), 5 μL of each restriction enzyme (NheI-HF, BamHI-HF and SalI-HF) can be added to each tube and digested for further 24 h at 37°C to ensure proper linearization.

### Problem 4

No binding is detectable after initial rounds of bead sorting and error prone PCR (related to step 26.e).

### Potential solution

Make sure that the display level of the library is sufficient. Continue with 2–3 flow cytometric positive sorts. If there is still no signal, go back to the epPCR library and continue with bead selections to enrich low affinity binders from the library (followed by another round of epPCR to improve affinities) or repeat selections with higher antigen concentrations. Generally, the success of selections is highly dependent on the quality of the library and of the antigen. Low diversity libraries may not contain any binders to a selected antigen.

## Resource availability

### Lead contact

Further information and requests for resources and reagents should be directed to and will be fulfilled by the lead contact, Michael W. Traxlmayr (michael.traxlmayr@boku.ac.at).

### Technical contact

Questions about the technical specifics of performing the protocol should be directed to and will be answered by the technical contact, Delia Sumesgutner (delia.sumesgutner@boku.ac.at).

### Materials availability

The scaffold libraries and pCTCON2V plasmid will be made available by the lead contact upon request.

### Data and code availability

This paper does not report original code.

## Acknowledgments

This work was supported by the Austrian Science Fund (FWF Project W1224 – Doctoral Program on Biomolecular Technology of Proteins – BioToP), the Federal Ministry for Digital and Economic Affairs of Austria, and the National Foundation for Research, Technology and Development of Austria to the Christian Doppler Research Association (Christian Doppler Laboratory for Next Generation CAR T Cells). The SH800S cell sorter and the CytoFLEX were kindly provided by the EQ-BOKU VIBT GmbH, and the project was supported by the BOKU Core Facility Biomolecular & Cellular Analysis.

## Author contributions

Conceptualization, M.W.T.; investigation, M.D.; writing – original draft, M.D. and D.S.; visualization, C.U.Z.; writing – review and editing, M.D., D.S., C.U.Z., and M.W.T.

## Declaration of interests

M.W.T. receives funding from Miltenyi Biotec.
